# The Influence of Polyethyleneimine’s Molecular Weight on the Physical, Chemical, and Biological Properties of Chitosan–Polyethyleneimine Carbon Dots and In Vitro Performances

**DOI:** 10.3390/mi17040501

**Published:** 2026-04-20

**Authors:** Sahin Demirci, Mehtap Sahiner, Selin S. Suner, Nurettin Sahiner

**Affiliations:** 1 Department of Food Engineering, Faculty of Engineering, Istanbul Aydin University, Florya Halit Aydin Campus, 34153 Istanbul, Turkey; sahindemirci@gmail.com; 2 Department of Bioengineering, Faculty of Engineering, Canakkale Onsekiz Mart University, Terzioglu Campus, 17100 Canakkale, Turkey; sahinerm78@gmail.com; 3 Department of Chemistry, Faculty of Sciences, Canakkale Onsekiz Mart University, Terzioglu Campus, 17100 Canakkale, Turkey; sagbasselin@gmail.com; 4 Department of Bioengineering, U.A. Whittaker College of Engineering, Florida Gulf Coast University, Fort Myers, FL 33965, USA; 5 Department of Chemical Engineering, Faculty of Engineering, Canakkale Onsekiz Mart University, Terzioglu Campus, 17100 Canakkale, Turkey

**Keywords:** carbon (quantum) dots/Cdots, N-enriched carbon dots, chitosan CDs, biocompatible Cdots, light-induced antibacterial activity

## Abstract

This study reports the effect of polyethyleneimine’s molecular weight (PEI, Mn: 1200, 10,000, and 60,000 g/mol, denoted as PEI_1.2_, PEI_10_, and PEI_60_) on the physical, chemical, and biological characteristics of carbon dots (Cdots) derived from chitosan (Chi) and PEI (Chi-PEI Cdots). The size of Chi Cdots was 41.5 ± 6.1 nm, which increased to 50.9 ± 5.9, 71.4 ± 4.2, and 93.3 ± 7.4 nm with the preparation of Chi-PEI_1.2_, Chi-PEI_10_ and Chi-PEI_60_ Cdots. The fluorescence properties and quantum yield% values of the Cdots prepared from Chi, PEI_1.2_, PEI_10_, and PEI_60_ and their corresponding biopolymeric Chi-PEI Cdots were compared. A higher quantum yield of 26 ± 1.6% was observed for Chi-PEI_1.2_. This decreased with the increasing molecular weight of PEI and was calculated to be 15 ± 1.9% for Chi-PEI_60_. All the prepared bare Chi, bare PEI and corresponding bipolymeric Chi-PEI Cdots were observed to be nonhemolytic up to a 1 mg/mL concentration. Lower cytotoxic properties were observed for Chi-PEI Cdots on L929 fibroblast cells compared to their corresponding bare forms. Higher cell viability was observed for Chi-PEI_1.2_ Cdots with 95% viability in the presence of a 1000 µg/mL concentration. The antibacterial activity of the prepared Cdots against pathogens such as *E. coli*, *K. pneumoniae*, *S. aureus*, *B. subtilis*, and *C. albicans* was investigated and compared. Lower MIC and MBC values were determined for Chi-PEI_10_ against *C. albicans* with values of 12.5 and 50 mg/mL, respectively. Although the antibacterial properties of Chi-PEI Cdots were less strong than those of bare Cdots derived from individual Chi and PEI molecules, their light-induced antibacterial activities were found to be better.

## 1. Introduction

Carbon dots (Cdots) are highly promising carbon-based nanostructures with low toxicity, excellent biocompatibility, strong photoluminescence, chemical stability, water solubility, and ease of surface modification [1,2]. These unique properties enable their broad application in bioimaging, drug delivery, biosensing, and gene delivery systems [3]. In particular, Cdots have attracted significant attention in drug delivery and biomedical applications as multifunctional platforms allowing sensing and real-time tracking, as well as for their potential roles as antimicrobial, anticancer, and neuroprotective agents, highlighting their versatility in both therapeutic and diagnostic applications [4]. According to the literature, Cdots have been extensively employed in a wide range of analytical and sensing applications, such as glucose sensing, Fe(III) determination, ascorbic acid detection, and Cu(II) quantification [5,6,7].

Chitosan (Chi), a naturally derived biopolymer, is a linear polysaccharide obtained by acetylation of chitin [8]. It has a structural unit that provides unique biodegradability, structural integrity, hydrophilicity, gel formation capability, and controlled drug release [9]. Chi offers benefits beyond its renewable nature, including a high nitrogen content and various functional groups like acetamido, amino, and hydroxyl groups [10]. It is widely used in the synthesis of carbon dots owing to its biocompatibility and abundance of functional amino groups [11]. Chi-based carbon dots have been extensively synthesized and investigated for multifunctional applications, including environmental pollutant detection, active food packaging, biosensing, and biomedical imaging [12].

Polyethylene imine (PEI) is a synthetic, water-soluble, nitrogen-rich polymer containing an ethylene amine repeating unit [13]. PEI exhibits antibacterial properties [14,15,16]. Previous studies reported the use of PEI for the surface modification and coating of Cdots [17,18]. In parallel, PEI plays a crucial role in nanoparticle functionalization and intracellular delivery processes due to its high density of amine groups and strong cationic nature [19,20]. PEI fluorescent Cdots were used to detect Co^2+^ [21].

Thakur et al. reported chitosan–PEI-based carbon dots using PEI with molecular weights of 2 kDa and 25 kDa in the presence of chitosan and complexed with plasmid DNA and miRNA as polyplexes to prevent serum-induced degradation [22]. Following that, the constructs’ decomplexation, serum stability, and mammalian cell viability were investigated, as well as their in vitro gene transfection capability and so on [22]. In a separate study, Chi-PEI Cdots were synthesized using microwave, hydrothermal, and microwave + hydrothermal synthesis techniques using PEI with an MW of 20,000 g/mol and chitosan with MWs ranging from 190,000 to 310,000 Da, and their cytotoxicity, hemolytic activity, and antibacterial properties were evaluated [23]. Despite many advantages, the systematic investigation of how PEI MW affects the physicochemical properties and biological performance of Chi–PEI Cdots remains unexplored. Existing studies primarily focus on the presence of PEI rather than the impact of its MW variations, leaving a critical gap in understanding structure–property–function relationships in these nanomaterials.

This study aims to comprehensively investigate the influence of PEI MW on the physical, chemical, and biological properties of Chi–PEI-based Cdots, with a particular emphasis on their in vitro biological performance. By elucidating the relationship between PEI MW and Cdot characteristics, this research provides valuable insights into rational design and optimization of Cdot formulations for biomedical applications.

## 2. Materials and Methods

### 2.1. Materials

Chitosan (Chi, medium molecular weight; Sigma Aldrich, St. Louis, MO, USA), branched polyethyleneimine (PEI_1.2_, Mn: 1200, 50% in water; Sigma Aldrich, St. Louis, MO, USA; PEI_10_, Mn: 10,000, 50% in water; Sigma Aldrich, Taufkirchen, Germany; PEI_60_, Mn: 60,000, 50% in water; Sigma Aldrich, Taufkirchen, Germany) and acetic acid (99.8–100.5%; Sigma Aldrich, Darmstadt, Germany) were used to synthesize Chi, PEI, and chi-PEI Cdots. *Escherichia coli* (*E. coli*, ATCC 8739; KWIK-STIK, Microbiologics, Saint Cloud, MN, USA), *Klebsiella pneumoniae* (*K. pneumoniae*, ATCC 70,0603; KWIK-STIK, Microbiologics, Saint Cloud, MN, USA), *Staphylococcus aureus* (*S. aureus*, ATCC 6538; KWIK-STIK, Microbiologics, Saint Cloud, MN, USA), *Bacillus subtilis* (*B. subtilis*, ATCC 6633; KWIK-STIK, Microbiologics, Saint Cloud, MN, USA), and *Candida albicans* (*C. albicans*, ATCC 10,231; KWIK-STIK, Microbiologics, Saint Cloud, MN, USA) were employed in antimicrobial studies of the related Cdots. These bacteria were grown in nutrient broth (NB; Merck, Darmstadt, Germany) as a liquid medium and using nutrient agar (NA; Condolab, Madrid, Spain) as a solid medium. In the toxicity analysis of the nanogels, L929 fibroblast cells (mouse C3/An connective tissue) were supplied by a local vendor (SAP Institute, Ankara, Turkey), and the culture medium used was Dulbecco’s modified Eagle’s medium (DMEM, containing 4.5 g/L glucose, 3.7 g/L sodium pyruvate, and 0.5 g/mL L-Glutamine; PanBiontech GmbH, Aidenbach, Germany) enriched with fetal bovine serum (FBS; PanBiontech GmbH, Aidenbach, Germany) and antibiotic solution (100 IU/mL penicillin/100 μg/mL streptomycin; PanBiontech, GmbH, Aidenbach, Germany). Trypsin/EDTA (0.25% Trypsin/0.02% EDTA; PanBiontech, GmbH, Germany), trypan blue solution (0.5%; Biological Industries, Haifa, Isreal), 3-(4,5-dimethylthiazol-2-yl)-2,5-diphenyltetrazolium bromide (MTT agent; neoFroxx GmbH, Einhausen, Germany) and dimethyl sulfoxide (DMSO, 99.9%; Carlo-Erba GmbH, Emmendingen, Germany) were used as received. Deionized water was obtained with a Millipore-Direct Q UV3 (Millipore Corp., Molsheim, France) at 18.2 M·Ω·cm.

### 2.2. Synthesis of Chi-PEI Based Cdots

The synthesis of Chi, PEI (1.2, 10, and 60), along with their respective Chi-PEI carbon dots (Cdots), was accomplished through a single-step hydrothermal method [24]. In the preparation of Chi Cdots, 0.5 g of Chi was dissolved in 15 mL of 0.5 M acetic acid, which was subsequently transferred into a 25 mL Teflon-lined autoclave. The autoclave was then placed in a furnace, where it was heated to 240 °C at a rate of 10 °C/min, maintaining this temperature for three hours. A similar procedure was employed for the synthesis of all other PEI Cdots. To prepare Chi-PEI Cdots, 0.5 g Chi and 0.5 g of the related PEI species (1.2, 10, or 60) were placed in 15 mL 0.5 M acetic acid and stirred for 1 h. Finally, this was transferred into a 25 mL Teflon-lined autoclave and placed in a furnace for the hydrothermal synthesis procedure mentioned above. Following synthesis, the resultant solutions underwent centrifugation at 10,000 rpm for five minutes to facilitate the precipitation of larger carbon particles. Following the elimination of larger carbon particles, the transparent Cdot solutions were transferred into a dialysis membrane (molecular weight cut-off ≥ 12,000 Da) for the purification for the removal of contaminants. The dialysis process was performed for 24 h against distilled water, and the wash water was replaced every 8 h. The transparent supernatants were then collected and precipitated in acetone, followed by drying with a heat gun. All formulations of Cdots, including Chi, PEI_1.2_, PEI_10_, PEI_60_, Chi-PEI_1.2_, Chi-PEI_10_, and Chi-PEI_60_, were stored at ambient temperature and shielded from light exposure.

### 2.3. Characterization of Chi-PEI Cdots

The Fourier transform infrared (FT-IR) spectra of the synthesized Cdots based on Chi-PEI were recorded using a Nicolet iS10 spectrometer (Thermo, Waltham, MA, USA) with a wavelength range from 4000 to 650 cm^−1^ and a resolution of 4 cm^−1^, employing the attenuated total reflectance (ATR) technique.

XRD patterns of the BPEI CDs were collected utilizing an X’Pert Pro MPD diffractometer (PANalytical, Almelo, The Netherlands), which was supplied with CuKα radiation and the X’Celerator detector for the diffracted beam. The XRD data were acquired in a Bragg Brentano (θ/θ) vertical arrangement, running in flat reflection mode, across a range of 5° to 70° (2θ) in increments of 0.02° 2θ, with a counting time of 1 s per step. The X-ray tube functioned at 45 kV and 40 mA, and a 1/2° divergence slit, a 0.04 rad soller slit, and a 10 mm fixed mask were set in the direction of the incident beam. The High Score Plus (v.4.6.0) software provided peak recognition and automatic search-match for the evaluation of the diffraction patterns.

The zeta potential and dynamic light scattering (DLS) analyses of the Chi-PEI based Cdots were performed using a Nanobrook OMNI (Brookhaven Instruments, Brookhaven, NY, USA), using solutions in 1 mM KNO_3_ for zeta potential and 10 mM KNO_3_ for DLS measurements at a concentration of 10 mg/mL.

The optical properties of the Chi-PEI based Cdots were examined using UV-Vis spectroscopy (Spectrum, Farmingdale, NY, USA) and fluorescence spectroscopy (Lumina, Thermo, Markham, ON, Canada) at room temperature.

### 2.4. Biocompatibility of Chi-PEI Cdots

#### 2.4.1. Hemocompatibility Assay of Chi-PEI Cdots Using Hemolysis and Blood Clotting Index

Blood specimens were obtained from healthy participants with the approval of the Human Research Ethics Committee at Canakkale Onsekiz Mart University (2011-KAEK-27/2022–2,200,063,689) and stored in hemogram tubes containing EDTA as an anticoagulant. The tubes were then gently inverted to guarantee thorough mixing of the blood. The hemocompatibility of Cdots was evaluated using hemolysis and blood coagulation assays, following the established protocols outlined in the pertinent literature [25].

#### 2.4.2. Cytotoxicity Evaluation of Chi-PEI Cdots Using MTT Assay on L929 Fibroblast Cells

A total of 10 mg of each type of synthesized Cdots was suspended in 10 mL of DMEM, which was enriched with 10% FBS and 1% antibiotics, and gently mixed to form uniform sample solutions at concentrations of 1 mg/mL. These solutions were subsequently diluted in DMEM to obtain various concentrations of 10, 50, 100, and 500 µg/mL. For the cell culture experiments, frozen L929/An2 fibroblasts, sourced from mouse C3/A connective tissue, were thawed at 37 °C and carefully placed in 15 mL tubes. The tubes were centrifuged at 100 *g* for 3 min, after which 3 mL of DMEM cell culture medium was added, and the mixture was transferred into 25 cm^3^ flasks. These flasks were incubated at 37 °C in a CO_2_ incubator, maintaining an environment of 5% CO_2_ and 95% air. The cytotoxic effects of the prepared Cdots were evaluated using L929 fibroblasts. Initially, the attachment of the cells was confirmed, and a cell suspension containing 1 × 10^4^ cells in DMEM was added to each well of a 96-well plate, followed by 24 h incubation at 37 °C. After this incubation, the old medium was removed, and samples at concentrations ranging from 50 to 1000 µg/mL were introduced into the wells for an additional 24 h incubation. The control group was treated with only DMEM medium. After the incubation period, the medium in the wells was discarded, and the wells were washed twice with phosphate-buffered saline (PBS). Subsequently, 100 µL of a 10-fold diluted MTT solution (tetrazolium bromide) was added to each well to assess the metabolic activity of viable cells, and the plate was incubated at 37 °C for 4 h in the dark. Finally, the MTT solution was removed, and 200 µL of dimethyl sulfoxide (DMSO) was added to dissolve the formazan crystals. The viability of the cells was quantified by measuring the absorbance of the 96-well plate using a plate reader microplate photometer (Multiskan™ FC, Thermo Fisher Scientific, Waltham, MA, USA) at a wavelength of 570 nm. The experiments were repeated three times.

### 2.5. Antibacterial Activity of Chi-PEI Cdots

#### 2.5.1. Microdilution Assay

The antibacterial and antifungal properties of Chi-PEI Cdots were evaluated using microtiter assays, adhering to established methodologies found in the existing literature [25]. Cultures of *Escherichia coli* (*E. coli*, ATCC 8739) and *Klebsiella pneumoniae* (*K. pneumoniae*, ATCC 700603) representing Gram-negative bacteria, along with *Staphylococcus aureus* (*S. aureus*, ATCC 6538) and *Bacillus subtilis* (*B. subtilis*, ATCC 6633) as Gram-positive bacteria and *Candida albicans* (*C. albicans*, ATCC 10231) as a yeast strain, were prepared in nutrient broth (NB) medium to achieve a McFarland standard of 0.5. These microbial suspensions were subsequently diluted to a concentration of 10 mg/mL in NB, and 100 µL of these samples was transferred into a 96-well plate. Before the experiment, the plate underwent sterilization under UV light (420 nm) for 3 min. Following this, 5 µL of the microorganism suspension was added to each well, while a negative control was established by adding only 5 µL of the bacteria or fungus to 100 µL of NB. The plates containing bacteria were incubated at 37 °C for 24 h, whereas the wells containing yeast were incubated at 25 °C for 48 h. After the incubation period, the bacterial or fungal suspension was introduced into the wells containing 10 mg/mL of Chi-PEI Cdots, and the bacterial growth inhibition% was assessed using a plate reader (Multiskan™ FC, Microplate Photometer) at a wavelength of 590 nm, with results compared against the negative control. Each test was conducted in triplicate, and the findings are reported along with standard deviations. The minimum inhibitory concentration (MIC) of the Chi-PEI Cdots was established as the lowest concentration at which no turbidity from bacterial or fungal growth is detected. To ascertain the minimum bactericidal or fungicidal concentration (MBC/MFC), 50 µL of the bacterial or fungal culture was inoculated onto solid media, specifically nutrient agar for bacteria and potato–dextrose agar for fungi, to evaluate the growth of microorganisms. The nutrient agar plates were incubated at 25 °C for 24 h, while the potato–dextrose agar plates for fungal strains were incubated for 48 h at the same temperature. MBC/MFC is defined as the minimum concentration of the material that achieves a 99.9% reduction in the growth of bacteria or fungi.

#### 2.5.2. Light-Activated Antibacterial Assay

The photodynamic antibacterial efficacy of Chi-PEI Cdots was evaluated against the Gram-negative bacterium *E. coli*, the Gram-positive bacterium *S. aureus*, and the yeast *C. albicans*, utilizing a UV-A light source (315–400 nm, 300 W; Ostram GmbH, Ultra vitalux, Munich, Germany) for comparison with conditions devoid of light. In this study, a concentration of 10 mg/mL of Chi-PEI Cdots was introduced into a bacterial/fungal culture solution calibrated to the McFarland 0.5 standard in NB. The experimental plates were subjected to incubation in both dark and UV-A light environments for a duration of 30 min. As a control, plates containing only the bacterial/fungal culture in NB were analyzed under identical conditions. Following the incubation period, the plates were assessed using a plate reader (Multiskan™ FC, Microplate Photometer) at a wavelength of 590 nm, and the results were compared with those for the negative control to ascertain the bacterial cell viability% in the presence of Chi-PEI Cdots under both light and dark conditions. Each test was conducted in triplicate, and the results are reported along with standard deviations.

## 3. Results and Discussion

### 3.1. Structural and Optical Comparison of Chi-PEI Cdots

**Figure 1:**
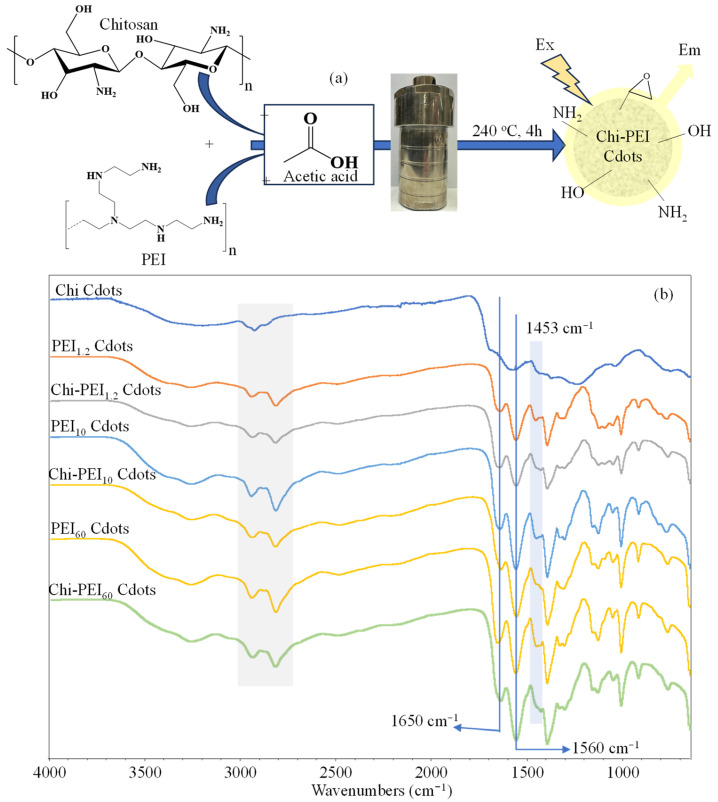
(**a**) Schematic representation of synthesis of Chi-PEI Cdots and (**b**) comparison of FT-IR spectra for prepared Chi, PEI, and Chi-PEI Cdots.

The synthesis of Cdots from Chi, PEI, and Chi-PEI was achieved using bottom-up approaches, as detailed in the literature with specific modifications. Figure 1a,b illustrate the main synthesis pathways and the FT-IR spectra corresponding to all synthesized Cdots, respectively. A total of seven formulation combinations were utilized to produce Cdot solutions, including Chi, PEI_1.2_, PEI_10_, and PEI_60_ Cdots and their combinations: Chi-PEI_1.2_, Chi-PEI_10_, and Chi-PEI_60_ Cdots. Previous studies reported the synthesis of Chi and PEI Cdots under hydrothermal conditions characterized by high temperature and pressure [23]. The mechanism proposed for the formation of Cdots from Chi or PEI chains involves hydrolysis of the precursor, followed by dehydration, polymerization, and condensation [23,26,27]. Moreover, the degree of deacetylation is a crucial property of Chi, as it is linked to the number of free amino groups that can be protonated in an acidic environment [28,29,30]. These free amino groups are essential for the characteristics of Chi. The functional groups in the Cdot structure are directly related to the initial materials employed. A higher deacetylation degree of Chi (90%) leads to increased availability of amino groups within the Chi matrix, which can be more easily incorporated into the Cdot core through covalent bonding, thus enhancing functionalization and improving quantum yield [31]. Additionally, the molecular weight of PEI significantly affects the physical and chemical properties of the resulting Cdots, as an increase in molecular weight results in a higher number of primary, secondary, and tertiary amines in the structure [32,33].

The FT-IR spectra for Chi Cdots, PEI Cdots and their corresponding Chi-PEI Cdot forms were compared in Figure 1b. It was clearly seen that all Cdots exhibit notable similarities. The most prominent peaks identified include C=O stretching peaks near 1650 cm^−1^ and N–H bending peaks at about 1560 cm^−1^. Also, the peaks around 1453 cm^−1^ for homo PEI Cdots were ascribed to CH_2_ bending, and they were slightly changed in the FT-IR spectrum of Chi-PEI Cdots due to there being fewer CH_2_ groups in the structure of Chi. Similarly, the intensity of aliphatic CH peaks around 2800–2900 cm^−1^ also decreased in the FT-IR spectrum of Chi-PEI Cdots compared to homo PEI Cdots. These small changes in FT-IR spectra give positive ideas about the successful synthesis of Chi-PEI based Cdots, but it was also clear that more evidence is required. Therefore, the DLS, XRD and fluorescence properties of prepared Cdots were compared.

The diameters of the Cdots were also determined via DLS measurements and summarized in Table 1. After conducting the measurements, the dimensions of the synthesized Cdots varied in relation to Chi and the differing molecular weights of PEI molecules.

The size of the Chi Cdots was determined to be 41.5 ± 6.1 nm, while the sizes for PEI_1.2_, PEI_10_, and PEI_60_ Cdots were 24.9 ± 5.9 nm, 48.7 ± 4.4 nm, and 64.4 ± 4.9 nm, respectively. The sizes of Chi-PEI Cdots varied in relation to the molecular weight of PEI, with larger sizes noted in comparison to individual Cdots derived from the precursors. Consequently, the average size of Chi-PEI_1.2_ Cdots was 50.9 ± 5.9, that of Chi-PEI_10_ Cdots was 71.4 ± 4.2, and that of Chi-PEI_60_ Cdots was 93.3 ± 7.4 nm. Importantly, recent research indicates that certain subclasses of carbon dots (Cdots), particularly carbonized polymer dots or surface-functionalized carbon dots, may exhibit noticeably larger apparent sizes, possibly reaching tens of nanometers due to aggregation effects or polymer shells [34]. This finding is consistent with earlier estimates and research suggesting that the size of synthesized Cdots tends to increase as the molecular weight of PEI increases. The increase in size observed with higher molecular weights can be attributed to the greater abundance of hydrophilic functional groups on the molecular surface, which in turn increases their tendency to absorb water and swell in aqueous conditions [35,36,37].

**Figure 2:**
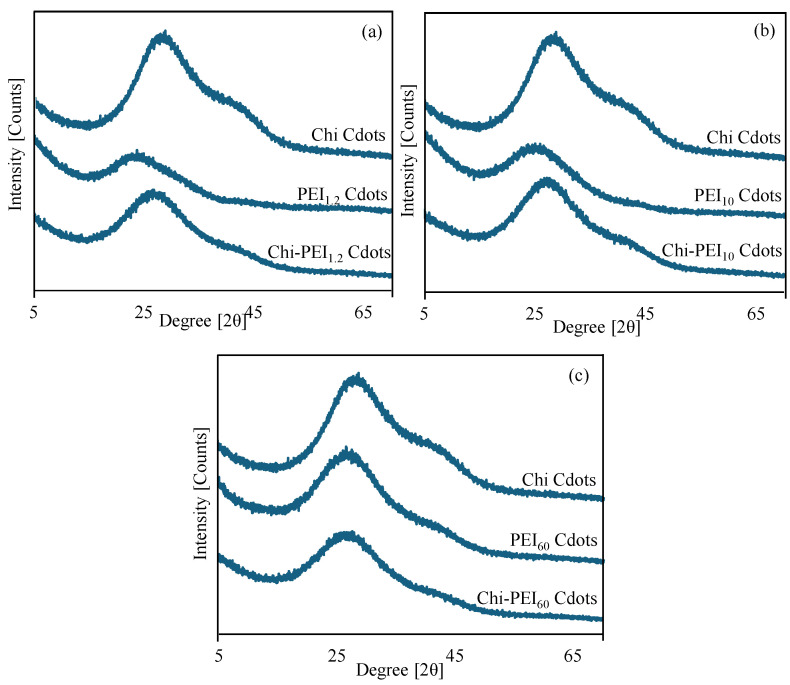
The comparisons of XRD patterns of Chi Cdots with those of (**a**) PEI_1.2_ and Chi-PEI_1.2_, (**b**) PEI_10_ and Chi-PEI_10_, and (**c**) PEI_60_ and Chi-PEI_60_ Cdots.

Furthermore, utilizing Chi and PEI, XRD analyses of Chi, PEI_1.2_, PEI_10_, PEI_60_, and their corresponding Cdot forms were performed, and the corresponding diffractograms are shown in Figure 2. The XRD patterns of Chi, PEI_1.2_, and Chi-PEI_1.2_ Cdots were compared in Figure 2a, and the peak at 2θ = 28.9° was assigned to the carbon phase, with crystallite sizes of 10 Å. For PEI_1.2_ Cdots, the carbon phase peak was slightly displaced to roughly 2θ = 24.2°, with a crystallite size of 7 Å. Chi-PEI_1.2_ Cdots exhibited a carbon phase peak at 2θ = 25.2° and crystallite sizes of 5 Å. On the other hand, the XRD patterns of Chi, PEI_10_, and Chi-PEI_10_ Cdots were also compared in Figure 2b. The carbon phase peak of PEI_10_ Cdots was clearly observed around 2θ = 28.9°, with 9 Å crystallite sizes, while that of Chi-PEI_10_ Cdots was observed around 2θ = 27.2°, with 8 Å crystallite sizes.

The XRD pattern comparisons of Chi, PEI_60_, and Chi-PEI_60_ Cdots are also shown in Figure 2c, and it was clearly seen that the carbon phase peaks for PEI_60_ and Chi-PEI_60_ Cdots were observed around 2θ = 26.8° and 26.7°, with crystallite sizes of 6 and 10 Å, respectively. In summary, the XRD patterns of Chi and homo PEI Cdots exhibit variations in relation to the observed carbon phase peaks. Conversely, the XRD patterns of Chi-PEI Cdots resemble those of Chi Cdots, except for Chi-PEI60 Cdots. This can be attributed to the fact that PEI with a high molecular weight exerts a greater influence than Chi. The determined crystallite sizes of Chi, PEI and Chi-PEI Cdots also showed differences, but no trend based on the molecular weight of the PEI chains used was observed.

**Figure 3:**
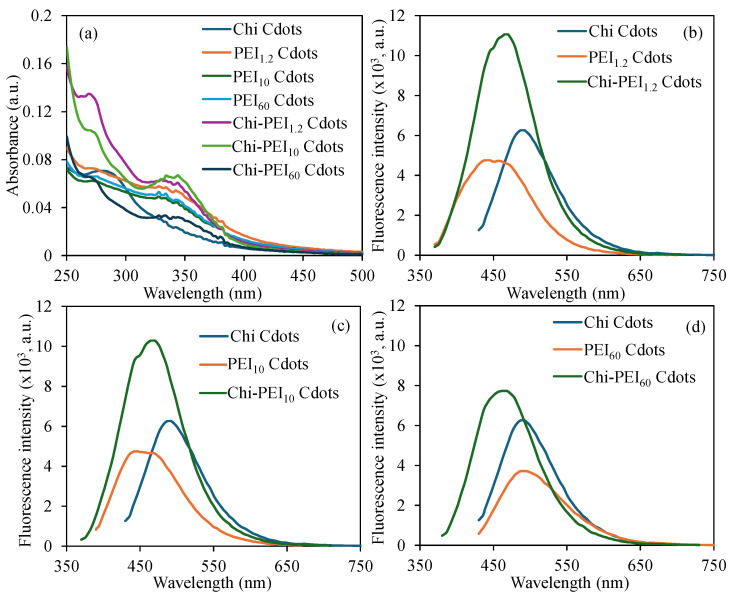
(**a**) UV-vis spectra for prepared Cdots, and comparison of fluorescence spectra of (**b**) Chi-PEI_1.2_, (**c**) Chi-PEI_10_, and (**d**) Chi-PEI_60_ Cdots with their corresponding bare Cdots.

Moreover, the UV-Vis spectra of the synthesized Cdots, shown in Figure 3a, revealed distinct absorption peaks indicative of different types of Cdots. In particular, Chi Cdots exhibited a peak at approximately 290 nm, while PEI Cdots, which varied in molecular weight, showed an absorption peak near 350 nm. Interestingly, Chi-PEI Cdots displayed both absorption peaks at around 290 nm and 350 nm, indicating a complex interaction among the components. The observed increase in the spectrum towards 250 nm can be attributed to the π-π* transition of the carbon–carbon double bond (C=C) occurring near 200 nm. This transition is a crucial element of the electronic structure of Cdots, highlighting their potential applications in photonics and materials science. Therefore, the fluorescence properties of the prepared Cdots were investigated. Firstly, the high density of amine groups from both Chi and PEI contributes to the effective surface passivation, thereby reducing non-radiative recombination pathways and enhancing fluorescence quantum yield [38,39]. Secondly, nitrogen doping introduced by PEI modifies the electronic structure of the carbon dots by creating new emissive surface states and trap sites, leading to tunable emission behavior [40]. Additionally, improved electron–hole recombination efficiency is achieved due to surface functionalization and heteroatom incorporation, which promotes radiative transitions [41]. Furthermore, intermolecular interactions between Chi and PEI influence π–π* transitions and surface defect states, contributing to the observed optical behavior [39].

The fluorescence spectra of Cdots prepared from Chi, PEI_1.2_, PEI_10_, and PEI_60_ in the presence of acetic acid were analyzed across excitation wavelengths ranging from 290 nm to 480 nm, as illustrated in Figure S1. In Figure S1a, Chi Cdots exhibits a significant redshift in the emission peak, moving from 425 nm to 515 nm as the excitation wavelength increases from 300 nm to 480 nm, with the peak fluorescence intensity of 6560 occurring at 475 nm when excited at 390 nm. Likewise, Cdots produced from PEIs of varying molecular weights demonstrated a similar redshift in emission wavelengths as the excitation wavelengths were altered from 300 to 500 nm. Notably, a higher molecular weight of PEI correlated with a more pronounced redshift in the maximum emission wavelength. The maximum emission wavelengths for Cdots from PEI_1.2_, PEI_10_, and PEI_60_ were recorded at 440 nm, 450 nm, and 490 nm, respectively, and the corresponding fluorescence intensities were 4760, 4730, and 3925 at excitation wavelengths of 360 nm, 380 nm, and 420 nm. The pertinent graphical representations are provided in Figure S1b–d. Furthermore, analogous investigations were performed on Chi-PEI Cdots, with the results depicted in Figure S2. Figure S2a,b reveal that the maximum emission wavelength is 470 nm at an excitation wavelength of 360 nm, with fluorescence intensities of 11,050 and 10,290 for Chi-PEI_1.2_ and Chi-PEI_10_ Cdots, respectively. Higher emission was also observed at 470 nm for Chi-PEI_60_ Cdots at a 7980 fluorescence intensity but at a 370 nm excitation wavelength. The redshift of emission wavelengths for Chi-PEI Cdots was also observed with the increase in excitation wavelengths from 300 to 480 nm.

Nitrogen-doped Cdots display a phenomenon referred to as aggregation-quenched fluorescence, which is marked by a notable decrease in fluorescence intensity as the concentration of these Cdots increases [42,43,44]. To explore this phenomenon, the concentrations of the synthesized Chi, PEI_1.2_, PEI_10_, and PEI_60_ Cdots were evaluated in aqueous solutions ranging from 25 to 0.2 mg/mL, as depicted in Figure S3. The measurements conducted at the designated excitation wavelength for each type of Cdot revealed peak emission values at a concentration of 12.5 mg/mL for Chi, PEI_1.2_, and PEI_10_ Cdots, while a concentration of 25 mg/mL was observed for Chi-PEI_60_. The fluorescence intensities for these Cdots decreased at concentrations both above and below these optimal levels. In addition, the effect of concentration on the fluorescence properties of Chi-PEI Cdots was analyzed, with the relevant graphs presented in Figure S4a–c for Chi-PEI_1.2_, Chi-PEI_10_, and Chi-PEI_60_, respectively. The findings indicate that the maximum fluorescence intensities were achieved at significantly lower concentrations compared to their single counterparts. Specifically, the concentrations that resulted in peak emission at the specified excitation wavelengths for the synthesized Chi-PEI Cdots were determined to be 0.78, 0.39, and 0.39 mg/mL for Chi-PEI_1.2_, Chi-PEI_10_, and Chi-PEI_60_ solutions, respectively. Moreover, the fluorescence intensities of Chi-PEI_1.2_, Chi-PEI_10_, and Chi-PEI_60_ Cdot solutions demonstrated reductions at both lower and higher concentrations.

The findings of this study reveal that fluorescence quenching associated with aggregation is markedly less pronounced in individual Cdots of Chi, PEI_1.2_, PEI_10_, and PEI_60_ when contrasted with their corresponding Chi-PEI_1.2_, Chi-PE_10_, and Chi-PEI_60_ variants. As the concentration of these Cdots increases, they are prone to aggregate formation, which enhances π-π stacking interactions in localized states [45]. This aggregation leads to a decrease in fluorescence intensity, attributable to the significant absorption of incident light by the fluorescent materials, resulting in spatial inhomogeneities in detection that ultimately reduce the emitted fluorescence [46,47]. Additionally, a secondary inner filter effect (sIFE) may manifest in solutions containing fluorescent materials, especially when there is considerable overlap between the absorption and emission spectra, potentially causing reabsorption of fluorescence emitted by the sample itself [48,49]. Interestingly, even at concentrations nearly 30 times lower, the fluorescence properties of Chi-PEI Cdots exceed those of their individual forms. This enhanced fluorescence behavior can be attributed to improved surface passivation arising from the high density of amine groups in both chitosan and polyethyleneimine, which reduces non-radiative recombination pathways [38,39]. In addition, nitrogen doping introduced by PEI contributes to the formation of new emissive surface states, further enhancing fluorescence properties [40]. Moreover, surface functionalization and heteroatom incorporation facilitate radiative electron–hole recombination processes [41]. The digital camera images of Cdots of Chi, PEI_1.2_, PEI_10_, PEI_60,_ and their corresponding Chi-PEI_1.2_, Chi-PE_10_, and Chi-PEI_60_ forms under sunlight and 254 nm and 366 nm UV light are shown in Figure S5. The prepared Cdots have no color under sunlight but emit a blue color under 366 nm UV light. Under 254 nm UV light, Chi-PEI Cdots exhibited emission, whereas no emission was observed for homo Chi and PEI Cdots.

In summary, the examination of fluorescence measurements for the relevant Cdots under optimal conditions—namely, excitation wavelength and concentration—is depicted in Figure 3b–d. The optimal parameters for all synthesized Cdots are summarized in Table 1. For the Chi Cdots illustrated in Figure 3b–d, peak emission was observed at a wavelength of 475 nm, demonstrating a fluorescence intensity of 6560 when excited at 390 nm, with the full width at half maximum (FWHM) of this emission peak estimated to be approximately 105 nm. Additionally, Figure 3b provides a comparative analysis of the fluorescence spectra for PEI_1.2_ and Chi-PEI_1.2_ Cdots, indicating that the maximum emission wavelength for PEI_1.2_ Cdots is 440 nm with a fluorescence intensity of 4760, while Chi-PEI_1.2_ Cdots exhibit a maximum emission wavelength of 470 nm and a fluorescence intensity of 11,050. The FWHM values for the fluorescence spectra of PEI1_.2_ and Chi-PEI_1.2_ Cdots were 114 nm and 96 nm, respectively. Figure 3c illustrates a comparative analysis of the fluorescence properties of Chi Cdots alongside bare PEI_10_ and Chi-PEI_10_ Cdots under optimal experimental conditions. The findings suggest that Chi-PEI_10_ Cdots display significantly enhanced fluorescence intensity in comparison to both Chi Cdots and PEI_10_ Cdots. Additionally, detailed examination of the FWHM values indicates that PEI_10_ Cdots possess an FWHM of 106 nm, while Chi-PEI_10_ Cdots exhibit a more refined FWHM of 98 nm. Figure 3d illustrates a comparative examination of the fluorescence properties of PEI_60_ and Chi-PEI_60_ Cdots, assessed under optimal experimental conditions. The findings reveal that Chi-PEI_60_ Cdots possess a higher fluorescence intensity compared to both Chi and PEI_60_ individual Cdots. The FHWM determinations for PEI_60_ and Chi-PEI_60_ Cdots were 104 nm and 98 nm, respectively. A detailed inspection of these FHWM values indicates that Chi, PEI_1.2_, PEI_10_, and PEI_60_ Cdots exhibit fluorescence characteristics over a wider spectral range in comparison to Chi-PEI Cdots. The FHWM value decreases as the molecular weight of PEI increases for single PEI Cdots, while the FHWM values for Chi-PEI Cdots remain relatively stable, regardless of the molecular weight of the PEI used.

Given that Chi, utilized in the synthesis of Chi-PEI-based Cdots which have intriguing optical properties, is one of the most prevalent natural antibacterial polymers and PEI is one of the foremost amine-based synthetic polymers, investigations were undertaken to assess the biomedical attributes, including blood compatibility and biocompatibility, as well as the potential ROS-mediated antibacterial properties, stemming from the optical characteristics of Chi-PEI Cdots.

### 3.2. Comparison of In Vitro Biomedical Properties of Chi-PEI Cdots

#### 3.2.1. In Vitro Blood Compatibility of Chi-PEI Cdots

**Figure 4:**
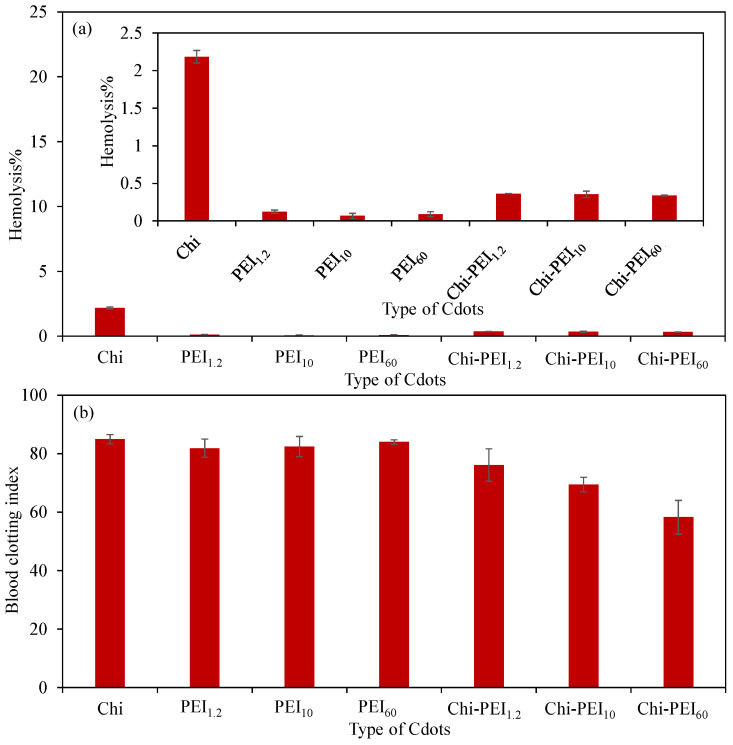
Hemocompatibility of Chi and PEI Cdots determined by in vitro (**a**) hemolysis% (inset figure is detailed) and (**b**) blood clotting% assays at 1 mg/mL sample concentrations.

The compatibility of materials with blood is a critical parameter, particularly in relation to their potential biomedical applications. It is essential to ascertain whether a material causes damage to red blood cells or interferes with the blood clotting mechanism, as these factors are pivotal when evaluating the blood compatibility of the material. Consequently, blood compatibility assessments were performed for Chi, PEI, and Chi-PEI Cdots, with the corresponding graphs illustrated in Figure 4a,b. In Figure 4a, the hemolysis% values recorded in the presence of the Cdots at a concentration of 1 mg/mL are compared. The literature evaluates that hemolysis% values < 2% signify nonhemolytic characteristics, with values ranging from 2 to 5% denoting slightly hemolytic characteristics and values > 5% indicating hemolytic characteristics [50]. As depicted in Figure 4a, the hemolysis% value for homo Chi Cdots was approximately 2%, whereas for homo PEI_1.2_, PEI_10_, and PEI_60_ Cdots, these values were below 0.2%. For Chi-PEI_1.2_, Chi-PEI_10_, and Chi-PEI_60_ Cdots, the hemolysis% values were around 0.35%. This indicates that the synthesized Chi Cdots are situated at the nonhemolytic/slightly hemolytic threshold with a concentration of 1 mg/mL, while the PEI-based and Chi-PEI Cdots are distinctly nonhemolytic at the same concentration. The marginally elevated hemolysis% values for Chi-PEI Cdots in comparison to homo PEI Cdots can be attributed to the incorporation of Chi into the structure.

The blood clotting index (BCI) serves as an indicator of the influence that various substances have on the blood clotting process. According to the existing literature, BCI values below 30% signify a strong clotting response (highly thrombogenic), values ranging from 30 to 60% indicate moderate clotting, values between 60 and 80% suggest mild or partial clotting, and values exceeding 80% reflect negligible clotting, implying no significant effect on the clotting mechanism [51,52]. In Figure 4b, a comparison of BCI values is presented for 1 mg/mL concentrations of Chi, PEI, and Chi-PEI Cdots. The BCI value for Chi-PEI Cdots was 84.9 ± 16% at a concentration of 1 mg/mL, while the values for PEI_1.2_, PEI_10_, and PEI_60_ Cdots were 81.9 ± 3.1, 82.4 ± 3.5, and 84.1 ± 0.7, respectively. Notably, for Chi-PEI Cdots, the BCI values exhibited a gradual decline as the molecular weight of PEI within the structure increased, with values of 76.1 ± 5.5, 69.5 ± 2.5, and 58.2 ± 5.8% for Chi-PEI_1.2_, Chi-PEI_10_, and Chi-PEI_60_ Cdots, respectively. The findings indicate that homo Chi and PEI Cdots displayed BCI values of around 80%, which aligns with the clotting effects reported in the literature [53,54]. Conversely, Chi-PEI Cdots demonstrated BCI values within the range of 60–80%, thereby influencing the blood clotting mechanism and resulting in mild or partial clotting [51,52,53]. Consequently, the synthesized Cdots are more appropriate for applications as composite materials in hemostatic agents, wound dressings, bleeding control patches, or surgical sealants, rather than for intravascular applications.

#### 3.2.2. In Vitro Cytotoxicity of Chi-PEI Cdots

**Figure 5:**
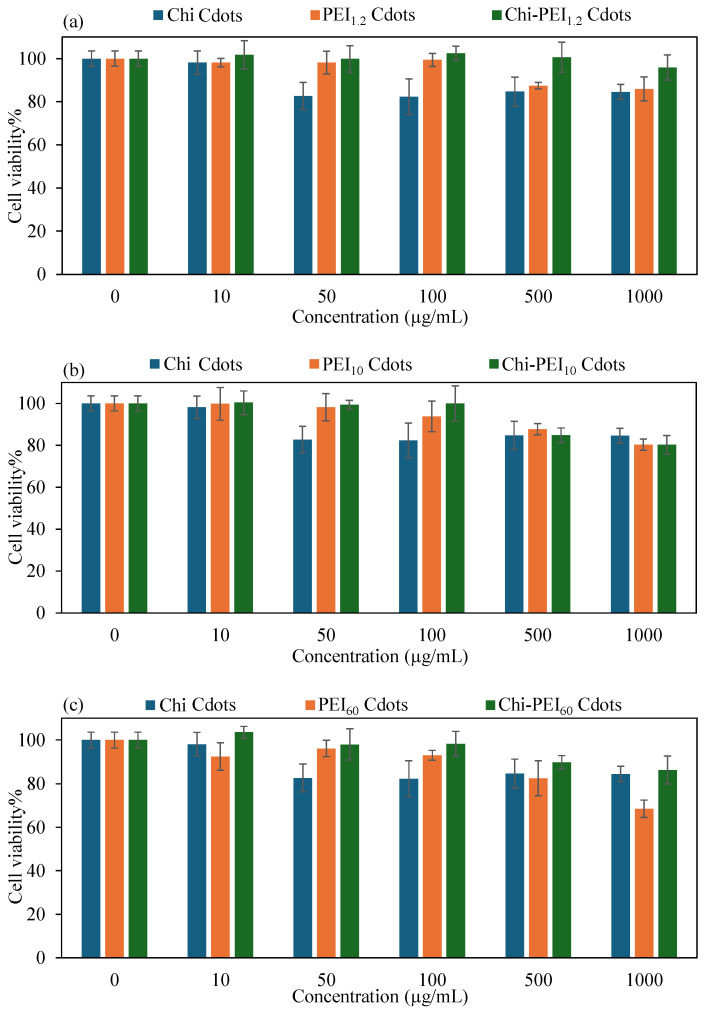
Concentration-dependent toxicity comparisons of Chi Cdots with (**a**) PEI_1.2_, Chi-PEI_1.2_, (**b**) PEI_10_, Chi-PEI_10_, and (**c**) PEI_60_, Chi-PEI_60_ Cdots against L929 fibroblast cells.

Another crucial factor for use in biomedical applications is the biocompatibility of materials. One of the most important aspects of the biomedical usefulness of materials is the absence of harmful effects after interactions with living cells. According to the literature, cell viability% values are classified as follows: >90% indicates that a material is non-cytotoxic (biocompatible), 80–89% is considered slightly cytotoxic (acceptable for most applications), 60–79% represents a mild cytotoxic effect (limited cell damage), 40–59% indicates moderate cytotoxicity (significant cell stress), and <40% indicates high toxicity [55]. Cell viability was investigated by evaluating the cytotoxicity of the synthesized Chi, PEI, and Chi-PEI Cdots on L929 fibroblast cells. Figure 5 shows the related comparative graphs and optic microscope images of the fibroblasts after being treated with 1000 μg/mL concentrations of the Chi, PEI_1.2_, PEI_10_, PEI_60,_ Chi-PEI_1.2_, Chi-PEI_10_, and Chi-PEI_60_ Cdots for a 24 h incubation time, as indicated in Figure S6. It is apparent from Figure S6 that the Chi and PEI-Chi Cdots have higher cell viabilities. The toxicities of homo PEI Cdots made from PEI with different molecular weights and Chi-PEI Cdots made of Chi and PEI are compared on L929 fibroblast cells in Figure 4, where Chi Cdots act as controls in each graph. Cell viability for Chi Cdots was over 98% at doses up to 50 µg/mL and over 80% at concentrations between 50 and 1000 µg/mL. In contrast, Figure 5a shows that for PEI_1.2_ Cdots, cell viability values of more than 98% were seen at concentrations up to 100 µg/mL, while values of over 85% were recorded at concentrations of 500 and 1000 µg/mL. Chi-PEI_1.2_ Cdots had more than 95% cell viability at doses as high as 1000 µg/mL.

The cytotoxicity characteristics of PEI_10_ and Chi-PEI_10_ Cdots are also shown in Figure 5b. At concentrations up to 100 µg/mL, PEI_10_ Cdots had cell viability values exceeding 93%, whereas at concentrations of 500 and 1000 µg/mL, the viability% values were 88 ± 2.7% and 80 ± 2.6%, respectively. Conversely, at a concentration of 100 µg/mL, Chi-PEI_10_ Cdots demonstrated over 99% cell viability, whereas values were greater than 80% for concentrations up to 1000 µg/mL. Figure 5c illustrates a comparison of the cytotoxic effects of PEI_60_ and Chi-PEI_60_ Cdots. In this context, the cell viability for PEI_60_ Cdots exceeded 93% at concentrations up to 100 µg/mL, 82 ± 8.0% at 500 µg/mL, and 68 ± 4.1% at 1000 µg/mL. For Chi-PEI_60_ Cdots, the cell viability was determined to be greater than 98% at 100 µg/mL and above 85% at 1000 µg/mL. To summarize, all Chi-PEI Cdots demonstrated non-toxicity at concentrations up to 50 µg/mL, maintaining cell viability above 90%, while homo PEI Cdots were also found to be non-toxic, with cell viability exceeding 90% at concentrations up to 100 µg/mL. For homo PEI Cdots, a minor reduction in cell viability was noted as the molecular weight of the PEI used for their synthesis increased, which is attributed to the heightened cytotoxicity associated with higher molecular weights of PEI [23,56,57]. In contrast, Chi-PEI Cdots exhibited superior cell viability% compared to homo Cdots at similar concentrations, suggesting that Chi-PEI Cdots possess greater potential for biomedical applications than homo Cdots. Chi-based Cdots are widely known for their excellent biocompatibility due to their innate origin and low toxicity profile, which is consistent with the high cell viability (>90%) also observed in this study at lower concentrations [58,59]. In contrast, nanomaterials based on PEI show toxicity depending on their molecular weight and concentration, mainly due to their high cationic charge density, which may damage cell membranes [60]. This accounts for the slight decrease in cell viability observed with higher concentrations and higher molecular weights of PEI. Importantly, the Chi–PEI Cdots demonstrated improved cytocompatibility compared to PEI Cdots, which can be attributed to the moderating effect of chitosan that reduces surface charge density and mitigates membrane damage [61].

### 3.3. Antibacterial Activity of Chi-PEI Cdots

**Figure 6:**
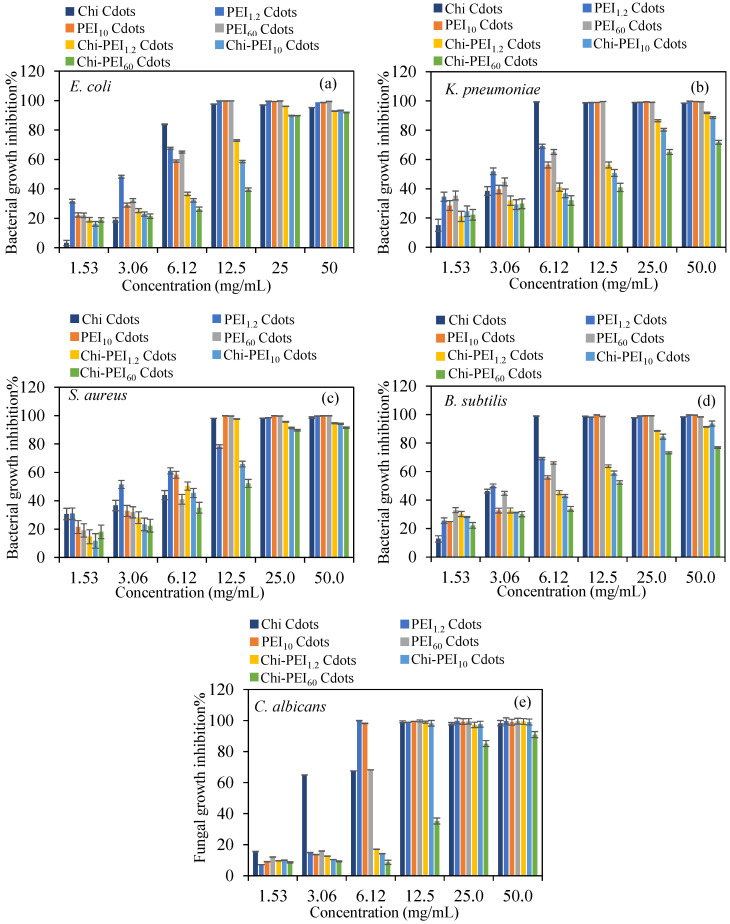
Concentration-dependent bacterial growth inhibition% values for prepared Chi-PEI Cdots against (**a**) *E. coli*, (**b**) *K. pneumoniae*, (**c**) *S. aureus*, (**d**) *B. subtilis*, and (**e**) *C. albicans* microorganisms.

In the fields of microbiology and medicine, antibacterial activity is crucial in addressing bacterial diseases that present significant threats to public health [62,63,64]. The capacity to suppress or eliminate pathogens is vital for controlling existing infections and preventing the emergence of resistant strains that may hinder treatment options [65,66]. Regardless of whether they are produced synthetically or sourced from nature, antibacterial drugs play a crucial role in the advancement of treatment strategies to control infectious diseases [67,68]. The efficacy of these substances directly influences clinical outcomes and public health, highlighting the importance of ongoing research and innovation. Moreover, the influence of antimicrobial properties extends beyond individual health, affecting broader public health initiatives and regulations. The antibacterial characteristics of Cdots have garnered significant interest recently, largely due to their unique physicochemical properties and applications in nanomedicine [69,70,71]. The efficacy of Cdots against bacteria is influenced by their size, surface charge, and functionalization [72]. Smaller Cdots often exhibit enhanced ability to infiltrate bacterial cells, even though positively charged Cdots have enhanced adhesion compared to negatively charged bacterial membranes [73,74]. Moreover, Cdots can augment their antibacterial properties by facilitating the production of reactive oxygen species (ROS) through light treatment, which have the potential to damage bacterial/fungal proteins and DNA [75,76]. Consequently, the antimicrobial efficacy of Chi-PEI Cdots and their homologous counterparts was evaluated using the microdilution method against pathogens such as *E. coli*, *K. pneumoniae*, *S. aureus*, *B. subtilis*, and *C. albicans* to ascertain and compare their capacities for inhibiting bacterial/fungal growth%. In Figure 6, for all Chi-PEI Cdots, increasing the concentration from 1.53 mg/mL to 50 mg/mL increased the bacterial/fungal growth inhibition% against all microorganisms.

Homo PEI_1.2_, PEI_10_, and PEI_60_ Cdots are more effective than Chi Cdots, except against *K. pneumoniae* and *B. subtilis* microorganisms, and all homo Cdots are more effective than their corresponding Chi-PEI Cdots. Above certain concentrations, microbial inhibition demonstrated remarkable efficacy, with values surpassing 90%. In some instances, the inhibition rates approached nearly 100%, underscoring the effectiveness of the concentrations used. This significant reduction in microbial proliferation indicates strong antimicrobial effects, suggesting that the applied concentrations were effective in disrupting the growth mechanisms of the bacteria/fungi. Therefore, the MIC and MBC/MFC values for the prepared Chi-PEI Cdots were determined and are compared in Table 2.

The MIC for *E. coli* was identified as 12.5 mg/mL for homo Chi, PEI_1.2_, PEI_10_, and PEI_60_ Cdots among Gram-negative bacteria. In contrast, the lowest MIC for *K. pneumoniae* was found to be 6.12 mg/mL for homo Chi Cdots, while a value of 12.5 mg/mL was recorded for the homo PEI Cdots. When comparing the MIC values for Gram-positive bacteria, the lowest MIC was exhibited by homo Chi, PEI_10_, PEI_60_, and Chi-PEI_1.2_ Cdots against *S. aureus* with a value of 12.5 mg/mL. For *B. subtilis*, the lowest MIC was recorded as 6.12 mg/mL for homo Chi Cdots. Additionally, the lowest MIC values against the yeast *C. albicans* were obtained for both homo PEI_1.2_ and PEI_10_ Cdots as 6.12 mg/mL.

Comparing the determined MBC values, the homo PEI Cdots exhibited the lowest MBC value of 12.5 mg/mL against *E. coli*. In contrast, the lowest MBC value for another Gram-negative bacterium, *K. pneumoniae*, was recorded for PEI_1.2_ Cdots, also at 12.5 mg/mL. The calculated MBC for *S. aureus* bacterium was 12.5 mg/mL for homo Chi, PEI_10_, PEI_60_, and Chi-PEI_1.2_ Cdots. The homo Chi, PEI_1.2_ Cdots, and Chi-PEI_1.2_ Cdots exhibited a lower MBC for the *B. subtilis* bacterium of 12.5 mg/mL. The results are particularly noteworthy since they show almost complete suppression of bacterial growth, in addition to a significant rate of inhibition. This highlights the potential of these treatments in treating bacterial infections, a critical problem in both clinical and environmental contexts, and suggests that the Cdots may target specific bacterial pathways or structures.

The Chi- and PEI-based Cdots were not designed for direct application or as antibiotic or antifungal agents. The synthesized Cdots are suitable for different surfaces as coatings and composite systems due to their higher MIC/MBC values that can prevent microorganisms from developing a habitat. These kinds of antimicrobial surface coatings can be included in wound dressing materials, implant surfaces, medical devices, or antimicrobial films [77] and as components of food packaging materials [78,79]. Although the Cdots prepared from Chi and PEI possess high minimum inhibitory concentration (MIC) values, they can rapidly and efficiently eradicate bacteria and fungi by generating reactive oxygen species (ROS) upon light exposure, such as ultraviolet (UV) or near-infrared (NIR) irradiation, due to their photodynamic antimicrobial capabilities [80].

**Figure 7:**
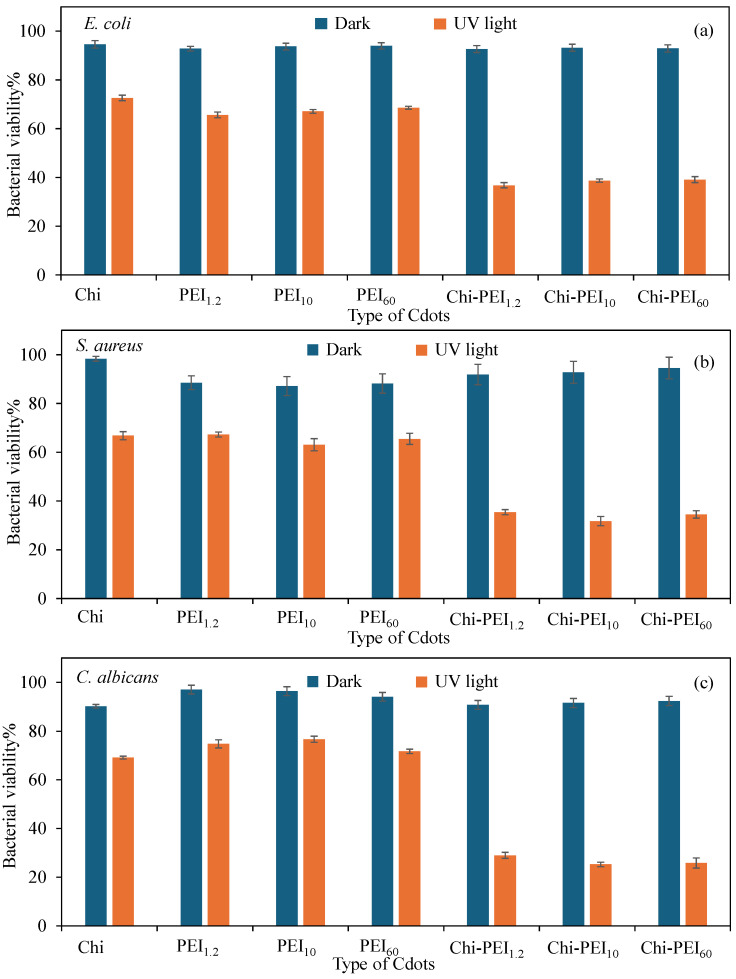
Comparison of light-induced antibacterial activity of Chi, PEI and Chi-PEI Cdots against (**a**) *E. coli*, (**b**) *S. aureus*, and (**c**) *C. albicans*.

At concentrations that closely correspond to their peak fluorescence intensity, the pertinent homo Cdots exhibit antibacterial properties. As illustrated in Figure S4, the fluorescence characteristics of Cdots derived from Chi-PEI diminish as the concentration increases, ultimately resulting in almost no fluorescence. It was proposed that the reduced antibacterial effectiveness of Chi-PEI Cdots is attributable to their quenched fluorescence characteristics at these concentrations, leading to a decrease in their capacity to produce ROS [81,82,83]. This observation aligns with the existing literature indicating a direct correlation between high fluorescence intensity, quantum yield values, and ROS activity [81,82,84]. As illustrated in the corresponding graph presented in Figure 7, the antibacterial activity of the pertinent homo and Chi-PEI Cdots activated by light was examined for solutions at a concentration of 1.56 mg/mL. The selected model microorganisms for this investigation included the Gram-positive *S. aureus*, the Gram-negative *E. coli,* and the fungus *C. albicans*. The bacterial viability% for *E. coli* at a concentration of 1.56 mg/mL under dark conditions after 30 min was determined to be 94.6 ± 1.5%, 92.8 ± 0.9%, 93.7 ± 1.3%, and 94.1 ± 1.3% in the presence of homo Chi, PEI_1.2_, PEI_10_, and PEI_60_ Cdots, respectively, as shown in Figure 7a. Conversely, the bacterial viability% for *E. coli* at the same concentration in the dark after 30 min in the presence of Chi-PEI_1.2_, Chi-PEI_10_, and Chi-PEI_60_ Cdots was 92.6 ± 1.4%, 93.2 ± 1.5%, and 92.9 ± 1.5%, respectively. The bacterial viability% for *E. coli* when exposed to the same concentration of homo Chi, PEI_1.2_, PEI_10_, and PEI_60_ Cdots was recorded as 72.6 ± 1.1%, 65.7 ± 1.2%, 67.1 ± 0.8%, and 68.6 ± 0.6%, respectively, during the antibacterial activity assessment conducted with 30 min of UV-A light exposure. The bacterial viability% for *E. coli* in the presence of Chi-PEI_1.2_, Chi-PEI_10_, and Chi-PEI_60_ Cdots under UV-A light after 30 min was 36.8 ± 1.1%, 38.6 ± 0.7%, and 39.1 ± 1.2%, respectively.

In a similar manner, the viability% of *S. aureus* bacteria under both dark and UV-A light conditions after 30 min was analyzed, as depicted in Figure 7b. As a result, the viability% values of *S. aureus* at concentrations of 1.56 mg/mL for Chi, PEI_1.2_, PEI_10_, and PEI_60_ Cdots were 98.3 ± 0.9%, 88.6 ± 2.8%, 87.1 ± 3.9%, and 88.2 ± 4.0%, respectively, in the dark after 30 min. In contrast, these values were 91.9 ± 4.2%, 92.7 ± 4.5%, and 94.6 ± 4.5%, respectively, when exposed to 1.56 mg/mL concentrations of Chi-PEI_1.2_, Chi-PEI_10_, and Chi-PEI_60_ Cdots in the dark after 30 min. Furthermore, the viability% of *S. aureus* in the presence of Chi, PEI_1.2_, PEI_10_, and PEI_60_ Cdots was 66.8 ± 1.7%, 67.3 ± 1.1%, 63.1 ± 2.5%, and 65.4 ± 2.3%, respectively, during antibacterial tests conducted under UV-A light after 30 min with the same concentration of Cdots. In the case of Chi-PEI_1.2_, Chi-PEI_10_, and Chi-PEI_60_ Cdots, the values were 35.4 ± 1.9%, 31.7 ± 1.9%, and 34.4 ± 1.5%, respectively.

Moreover, the antimicrobial properties of homo Chi, PEI_1.2_, PEI_10_, and PEI_60_ Cdots and their corresponding Chi-PEI Cdot forms activated by light were evaluated against the fungal species *C. albicans*, with results illustrated in Figure 7c. In a dark setting, the viability% for *C. albicans* when exposed to homo Chi, PEI_1.2_, PEI_10_, and PEI_60_ Cdots at a concentration of 1.56 mg/mL over a duration of 30 min was 90.1 ± 0.8%, 97.1 ± 1.8%, 96.4 ± 1.8%, and 94.1 ± 1.8%, respectively. In contrast, the viability% with Chi-PEI_1.2_, Chi-PEI_10_, and Chi-PEI_60_ Cdots was 90.7 ± 1.9%, 91.5 ± 2.1%, and 92.3 ± 1.9%, respectively. The viability% of *C. albicans* was 69.2 ± 1.9%, 74.8 ± 1.6%, 76.7 ± 1.3%, and 71.7 ± 1.2%, respectively, following 30 min of exposure to UV-A light with 1.56 mg/mL concentrations of Chi, PEI_1.2_, PEI_10_, and PEI_60_ Cdots. Under UV-A exposure for 30 min, the viability% of *C. albicans* with 1.56 mg/mL concentrations of Chi-PEI_1.2_, Chi-PEI_10_, and Chi-PEI_60_ Cdots was determined to be 28.9 ± 1.2%, 25.2 ± 0.9%, and 25.8 ± 2.1%, respectively. In summary, the light-activated antibacterial/fungal activities of Chi-PEI Cdots were more effective than their corresponding homo Cdots forms, which means a higher inhibition ability with respect to microbial growth. It is well known that photosensitive Cdots provide great advantages in photodynamic therapy in terms of microbial inhibition due to their reactive oxygen species (ROS) generation ability under UV or NIR light irradiation [85]. Fluorescent Cdots absorb photons of light and excite the singlet state to a triplet state. Then, two types of mechanism based on electron and energy transfer occur and produce superoxide and hydroxyl radicals and singlet oxygen-based ROS inside microbial cells via light exposure. Therefore, these reactive species damage multiple cellular components such as the membranes, DNA/RNA, and proteins of bacteria and fungi upon light exposure and trigger microbial cell death [86]. Numerous studies have shown that light-induced ROS production, which causes membrane rupture, protein oxidation, and DNA damage, is associated with Cdots’ antibacterial potency [86,87,88]. For example, research by Chen et al. found that ROS generation capability increased the antibacterial efficacy of Cdots upon light exposure [86]. Similarly, Huang et al. demonstrated that by promoting intracellular ROS generation during irradiation, doped Cdots caused significant bacterial inactivation [87]. Furthermore, Liang et al. showed that the antibacterial activity of Cdots was significantly influenced due to oxidative stress caused by ROS [88]. The photoinduced antimicrobial activity was directly dependent on the fluorescence ability of the Cdots. As can be seen in Figures S3–S5, the fluorescence intensity and quantum yield of the homo Cdots forms were significantly lower than those of the Chi-PEI Cdots at the same concentration. It was shown that Chi-PEI based Cdots are more photoactive materials with their high quantum yields and cause more damage to microorganisms. It should be noted, however, that this study did not test ROS directly, even though the increase in antibacterial activity upon UV-A light exposure is consistent with the ROS-mediated processes reported for Cdots.

## 4. Conclusions

In this study, effective development of Cdots based on Chi and PEI was carried out via hydrothermal methods, and the significance of PEI molecular weight was systematically clarified as a crucial factor influencing their physical, chemical, and biological efficacy. The increase in PEI molecular weight from 1200 to 60,000 increased the sizes of Chi-PEI Cdots almost 2-fold. The fluorescent intensity values of bare Chi Cdots were 6560, while the fluorescent intensities of PEI_1.2_, PEI_10_, and PEI_60_ Cdots were less than 5000. However, the fluorescent intensities of Chi-PEI Cdots were higher than those of the bare forms, which can be explained by a synergistic effect. The results indicate that the molecular weight of PEI acts as a significant tuning variable that regulates hemocompatibility, cytocompatibility, and antimicrobial properties. All synthesized bare Chi, bare PEI, and Chi-PEI Cdots demonstrated nonhemolytic properties up to a concentration of 1 mg/mL, thereby affirming their compatibility with blood. While both bare Chi and PEI Cdots exhibited elevated blood clotting indices, the addition of PEI to Chi Cdots resulted in a slight reduction in clotting propensity, with a decrease that was dependent on the molecular weight of PEI at higher values. Notably, Chi-PEI Cdots had enhanced cytocompatibility towards L929 fibroblasts in comparison to their bare versions, emphasizing the advantageous synergistic effect of polymer integration. Despite the fact that an increase in PEI molecular weight caused a slight reduction in cell viability, all formulations remained within acceptable thresholds for biocompatibility, highlighting their potential for use in practical applications. From an antimicrobial standpoint, bare Chi and PEI Cdots displayed more potent antimicrobial properties. When exposed to light irradiation, Chi-PEI Cdots had significantly improved antibacterial efficacy, which was directly linked to their ability to generate ROS. This transition from intrinsic antimicrobial effectiveness to photoactivated superiority highlights the functional benefits of the Chi-PEI Cdots for light-activated antimicrobial approaches. Elemental, XPS and EDX analyses quantitatively showed that the increase in the MW of the PEI used in the Cdot synthesis increased the N-doping extent. The increase in hydrodynamic diameter [80] and the decrease in fluorescence intensity [89,90] with increasing N-doping amounts and the increase in cytotoxicity with increasing PEI MW [91,92] are the few examples and are consistent with the literature. Collectively, these findings confirm that engineering PEI molecular weight serves as a logical design approach for customizing multifunctional carbon nanodots. The capacity to achieve a balance between hemocompatibility, cytocompatibility, and light-responsive antimicrobial properties makes Chi-PEI Cdots exceptionally promising candidates for cutting-edge biomedical applications, especially photodynamic antimicrobial therapy and infection-controlled wound management.

## Figures and Tables

**Table 1:** Comparison of sizes and fluorescent properties of prepared Chi and PEI Cdots.

Cdots	Diameter (nm)	Conc. (mg/mL)	λEx (nm)	λEm (nm)	Fluorescence Intensity	QY%
Chi	41.5 ± 6.1	12.5	390	475	6560	13 ± 0.8
PEI_1.2_	24.9 ± 5.9	12.5	360	440	4760	9 ± 0.5
PEI_10_	48.7 ± 4.4	12.5	380	450	4730	8 ± 0.6
PEI_60_	64.4 ± 4.9	25	420	490	3925	6 ± 0.5
Chi-PEI_1.2_	50.9 ± 5.9	0.78	360	470	11,050	26 ± 1.6
Chi-PEI_10_	71.4 ± 4.2	0.39	360	470	10,290	22 ± 1.3
Chi-PEI_60_	93.3 ± 7.4	0.39	370	470	7980	15 ± 1.9

**Table 2:** MIC and MBC/MFC values for Cdots against microorganisms.

Cdots	*E. coli*		*K. pneumoniae*		*S. aureus*		*B. subtilis*		*C. albicans*	
mg/mL	MIC	MBC	MIC	MBC	MIC	MBC	MIC	MBC	MIC	MFC
Chi	12.5	50	6.12	25	12.5	25	6.12	12.5	12.5	25
PEI_1.2_	12.5	12.5	12.5	12.5	25	25	12.5	12.5	6.12	50
PEI_10_	12.5	12.5	12.5	25	12.5	25	12.5	12.5	6.12	50
PEI_60_	12.5	12.5	12.5	25	12.5	12.5	12.5	50	12.5	25
Chi-PEI_1.2_	25	25	50	N.D	12.5	12.5	25	25	12.5	25
Chi-PEI_10_	25	25	50	N.D	25	25	25	25	12.5	50
Chi-PEI_60_	25	50	50	N.D	50	50	50	N.D	50	50

## Data Availability

The original contributions presented in the study are included in the article, further inquiries can be directed to the corresponding author.

## Supplementary Materials

The following supporting information can be downloaded at https://www.mdpi.com/article/10.3390/mi17040501/s1: Figure S1: Comparison of fluorescence properties of (a) Chi, (b) PEI_1.2_, (c) PEI_10_, and (d) PEI_60_ Cdots at different excitation wavelengths; Figure S2: Comparison of fluorescence properties of (a) Chi-PEI_1.2_, (b) Chi-PEI_10_, and (c) Chi-PEI_60_ Cdots at different excitation wavelengths; Figure S3: Comparison of fluorescence properties of (a) Chi, (b) PEI_1.2_, (c) PEI_10_, and (d) PEI_60_ Cdots at different concentrations at related excitations wavelengths; Figure S4: Comparison of fluorescence properties of (a) Chi-PEI_1.2_, (b) Chi-PEI_10_, and (c) Chi-PEI_60_ Cdots at different concentrations at related excitations wavelengths; Figure S5: The digital camera images of prepared Cdots under daylight and 254 and 366 nm UV light; Figure S6: Optical microscope images of the L929 fibroblast cells used as the negative control group and in the presence of 1000 µg/mL concentrations of the Chi, PEI_1.2_, PEI_10_, PEI_60_, Chi-PEI_1.2_, Chi-PEI_10_, and Chi-PEI_60_ Cdots for a 24 h incubation time.
