# Supplementary material for: The Influence of Polyethyleneimine’s Molecular Weight on the Physical, Chemical, and Biological Properties of Chitosan–Polyethyleneimine Carbon Dots and In Vitro Performances
Source: Micromachines (Basel). 2026 Apr 20;17(4):501. doi: 10.3390/mi17040501 (PMC13119019; doi:10.3390/mi17040501)
Supplement: Supplementary file 1 [file micromachines-17-00501-s001.zip › micromachines-4209640-supplementary.pdf]

micromachines

Supplementary Materials

The Influence of Polyethyleneimine's Molecular Weight on the Physical, Chemical, and Biological Properties of Chitosan–Polyethyleneimine Carbon Dots and In Vitro Performances

Sahin Demirci [1], Mehtap Sahiner [2], Selin S. Suner [3] and Nurettin Sahiner [4,5,*]

[1] Department of Food Engineering, Faculty of Engineering, Istanbul Aydin University, Florya Halit Aydin Campus, 34153 Istanbul, Turkey; sahindemirci@gmail.com
[2] Department of Bioengineering, Faculty of Engineering, Canakkale Onsekiz Mart University Terzioglu Campus, 17100 Canakkale, Turkey; sahinerm78@gmail.com
[3] Department of Chemistry, Faculty of Sciences, Canakkale Onsekiz Mart University Terzioglu Campus, Canakkale 17100, Turkey; sagbasselin@gmail.com
[4] Department of Bioengineering, U.A. Whittaker College of Engineering, Florida Gulf Coast University, Fort Myers, FL 33965, USA
[5] Department of Chemical Engineering, Faculty of Engineering, Canakkale Onsekiz Mart University, Terzioglu Campus, 17100 Canakkale, Turkey
* Correspondence: sahiner71@e-mail.com or nsahiner@fgcu.edu

Hemolysis Assay

Initially, a mixture of fresh blood and a 0.9% saline solution (SF) was prepared in a 1:1.25 ratio, combining blood with the saline. This mixture was thoroughly homogenized. Following this, 10 mg of Cdots were added to the tubes and suspended in the saline solution at concentrations of 1 mg/mL. Gradually, 200 μL of the diluted blood was introduced into the tubes containing the Cdots, and the mixture was incubated at 37.5 °C in a shaking bath for one hour. After the incubation, the Cdot suspensions were centrifuged at 500 g for five minutes, and the absorbance of the supernatant was measured using a UV–vis spectrophotometer at a wavelength of 542 nm to determine the amount of hemoglobin released. The hemolysis ratio was then calculated according to Equation (1).

Eq. (1) $\%\text{Hemolysis ratio} = \frac{(Asample - Anegative)}{(Apositive - Anegative)} x100 \qquad (1)$

Here, A_{sample} represents the absorbance of Cdots-containing blood solution. $A_{positive}$ and $A_{negative}$ are the absorbance of 200 μL diluted blood in 10 mL DI water and 0.2 mL diluted blood in 10 mL SF, respectively.

Blood Clotting Assay

In the method for evaluating blood coagulation, a 0.2 M aqueous solution of calcium chloride ($CaCl_2$) with a volume of 0.24 mL was added to 3 mL of freshly collected blood, which was then gently inverted twice to ensure adequate mixing. Following this, 0.27 mL of the resulting blood mixture was applied to 10 mg of Cdots within tubes, ensuring that the surfaces of the Cdots were completely covered. The tubes, now containing both the Cdots and the blood solution, were incubated at a temperature of 37.5 °C for 10 minutes. After the incubation period, 10 mL of deionized water was carefully introduced, and the mixture underwent centrifugation at a force of 100g for 40 seconds. The supernatant

obtained was then combined with 40 mL of deionized water and incubated once more at 37.5 °C for one hour. The absorbance of this final solution was measured using a UV–vis spectrophotometer at a wavelength of 542 nm, and the blood clotting index was calculated in accordance with Equation (2).

Eq.(2)

$$= \frac{(Asample+blood)}{(Ablood)} x100 \quad (2)$$

Here, ($A_{sample + blood}$) is the absorbance of blood solution contacted with the Cdots and A_{blood} is that of the blood solution (alone) diluted in 50 mL DI water.

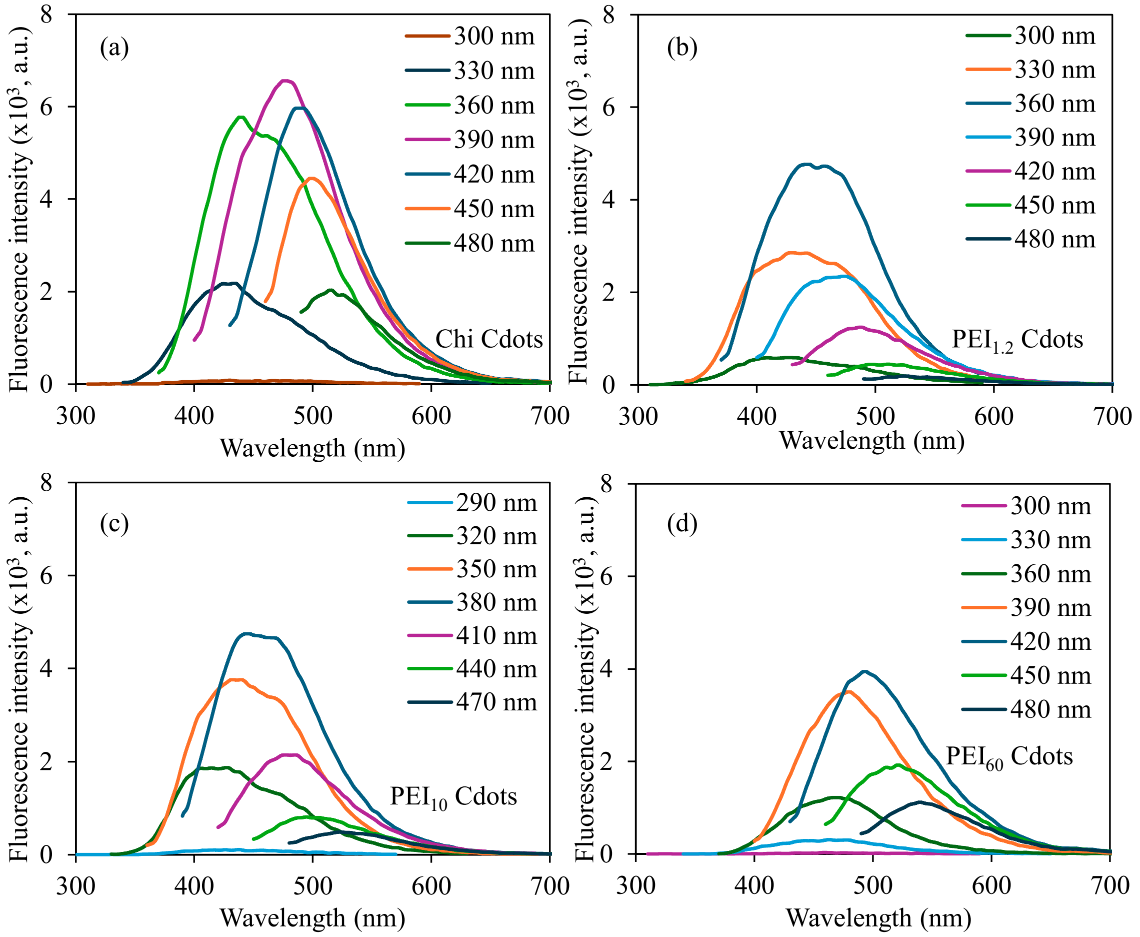

Figure S1. Comparison of fluorescence properties of (a) Chi, (b) $PEI_{1.2}$, (c) PEI_{10}, and (d) PEI_{60} Cdots at different excitation wavelengths.

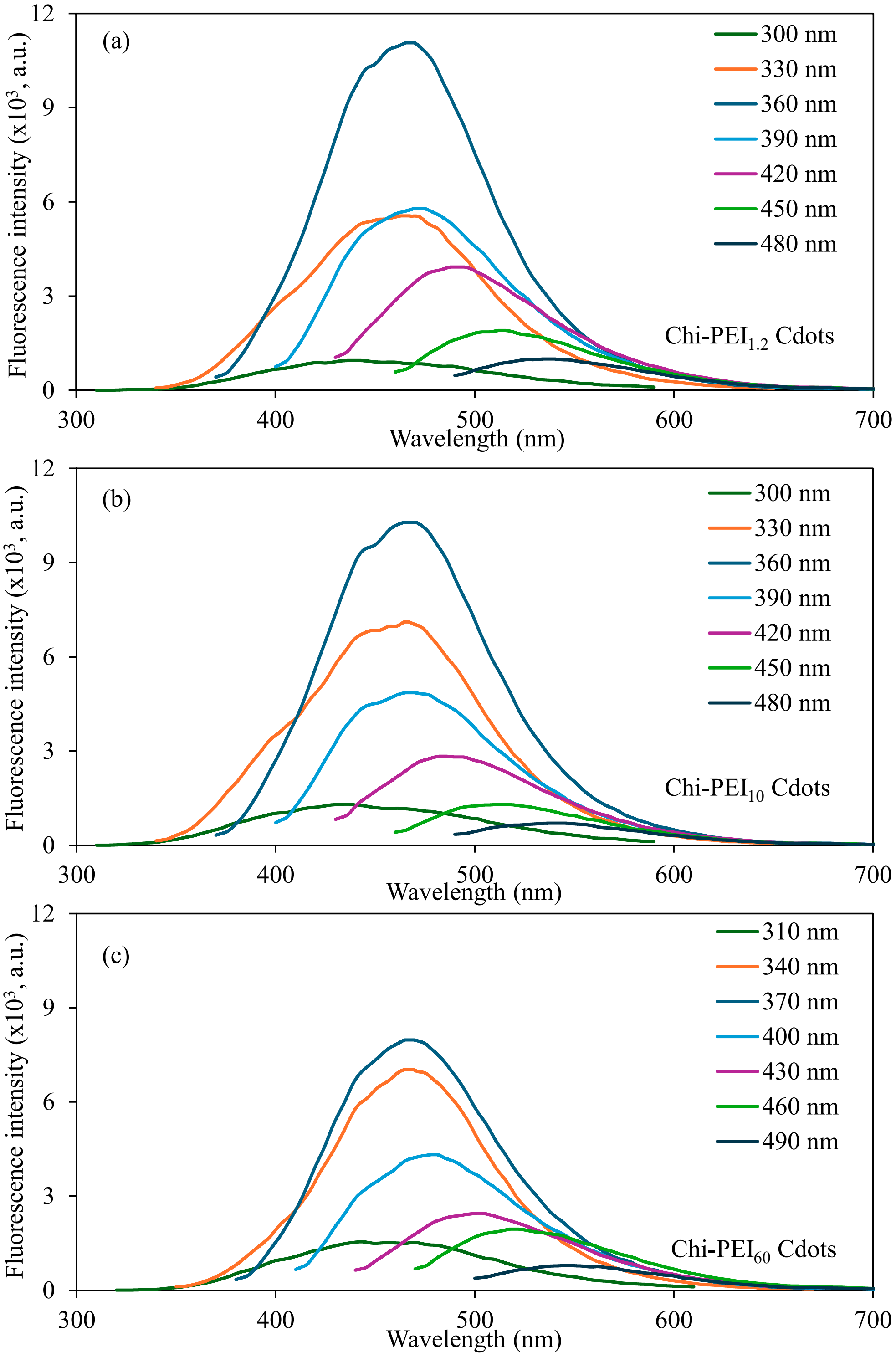

Figure S2. Comparison of fluorescence properties of (a) Chi-$PEI_{1.2}$, (b) Chi-PEI_{10}, and (c) Chi-PEI_{60} Cdots at different excitation wavelengths.

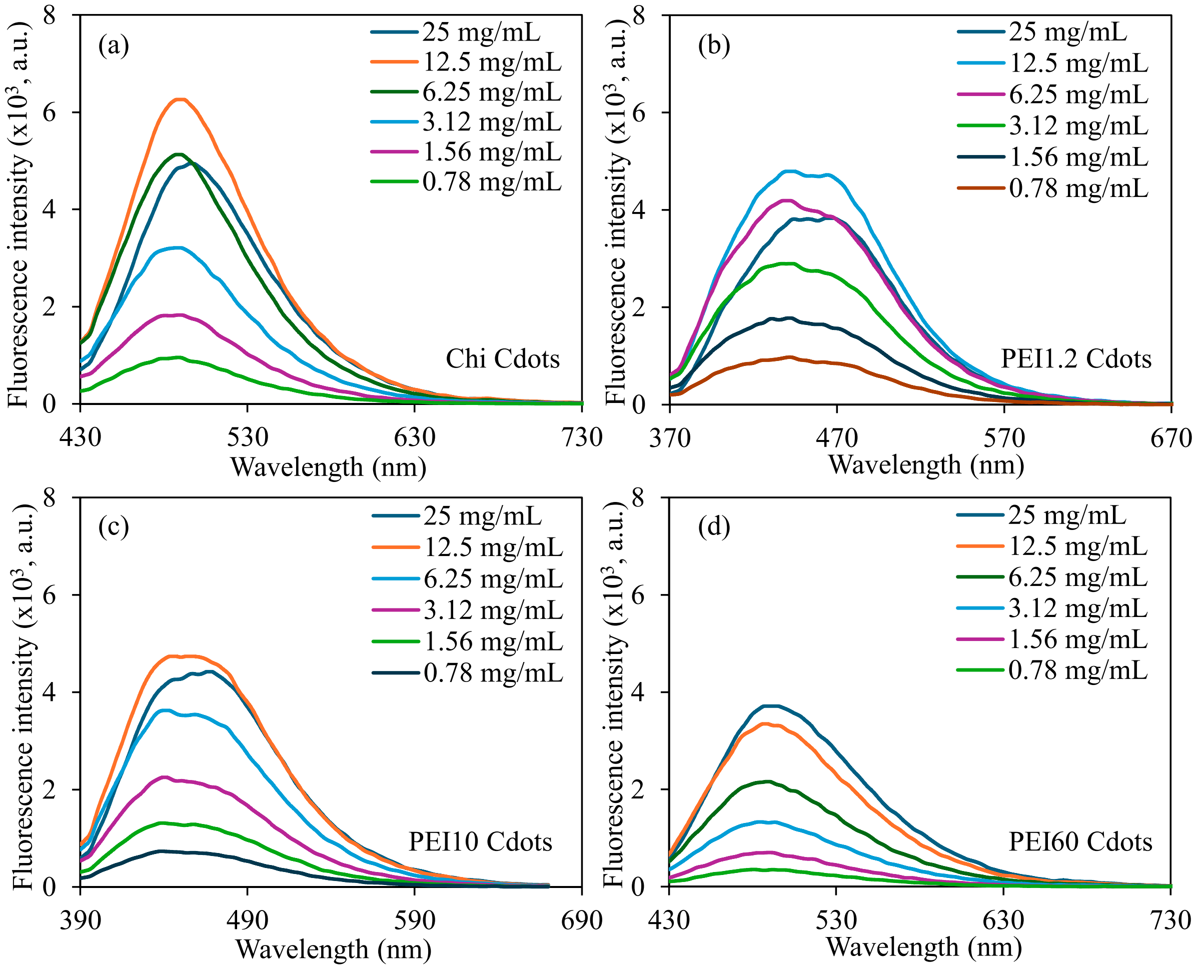

Figure S3. Comparison of fluorescence properties of (a) Chi, (b) $PEI_{1.2}$, (c) PEI_{10}, and (d) PEI_{60} Cdots at different concentrations at related excitations wavelengths.

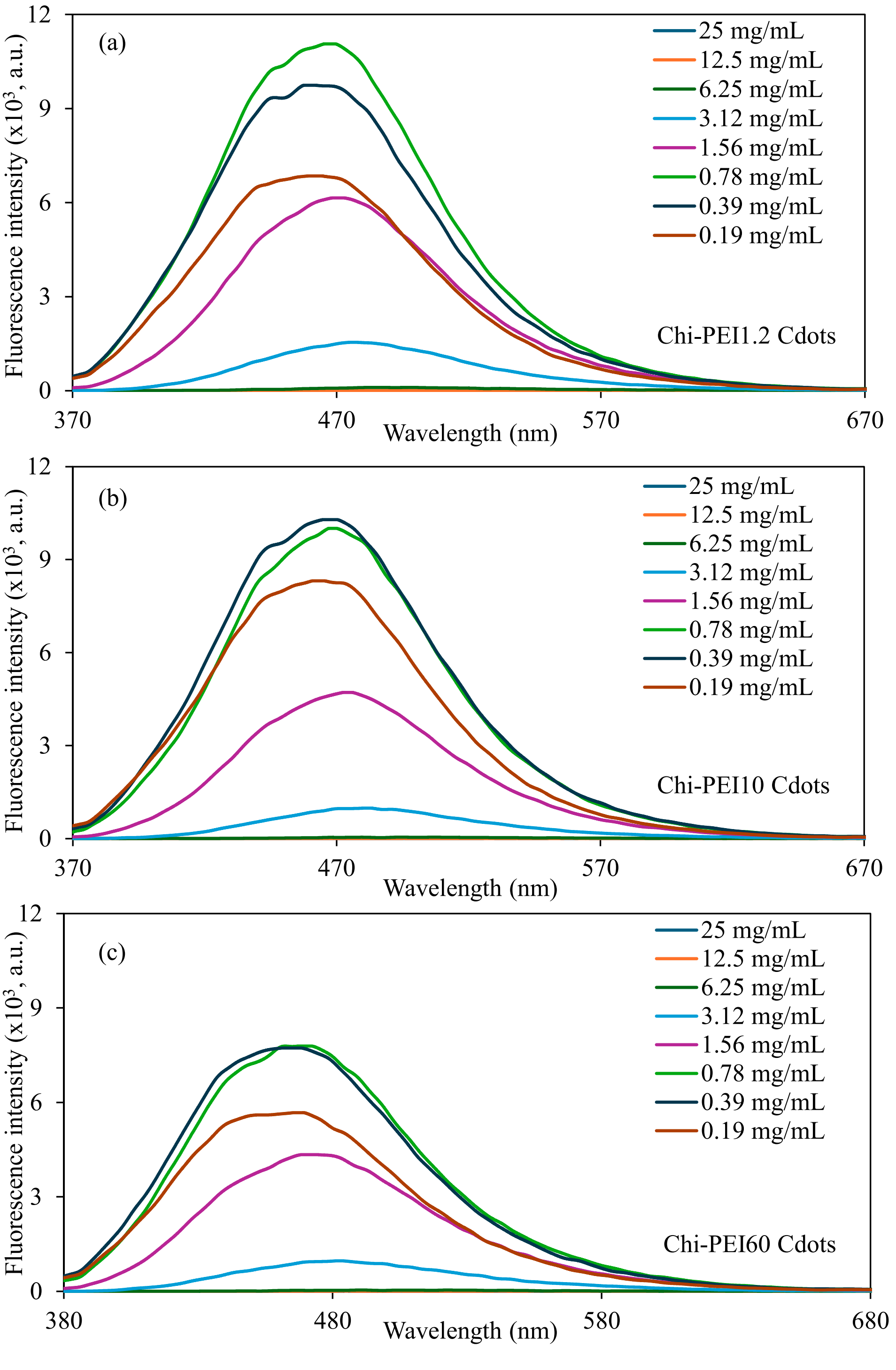

Figure S4. Comparison of fluorescence properties of (a) Chi-PEI$_{1.2}$, (b) Chi-PEI$_{10}$, and (c) Chi-PEI$_{60}$ Cdots at different concentrations at related excitations wavelengths.

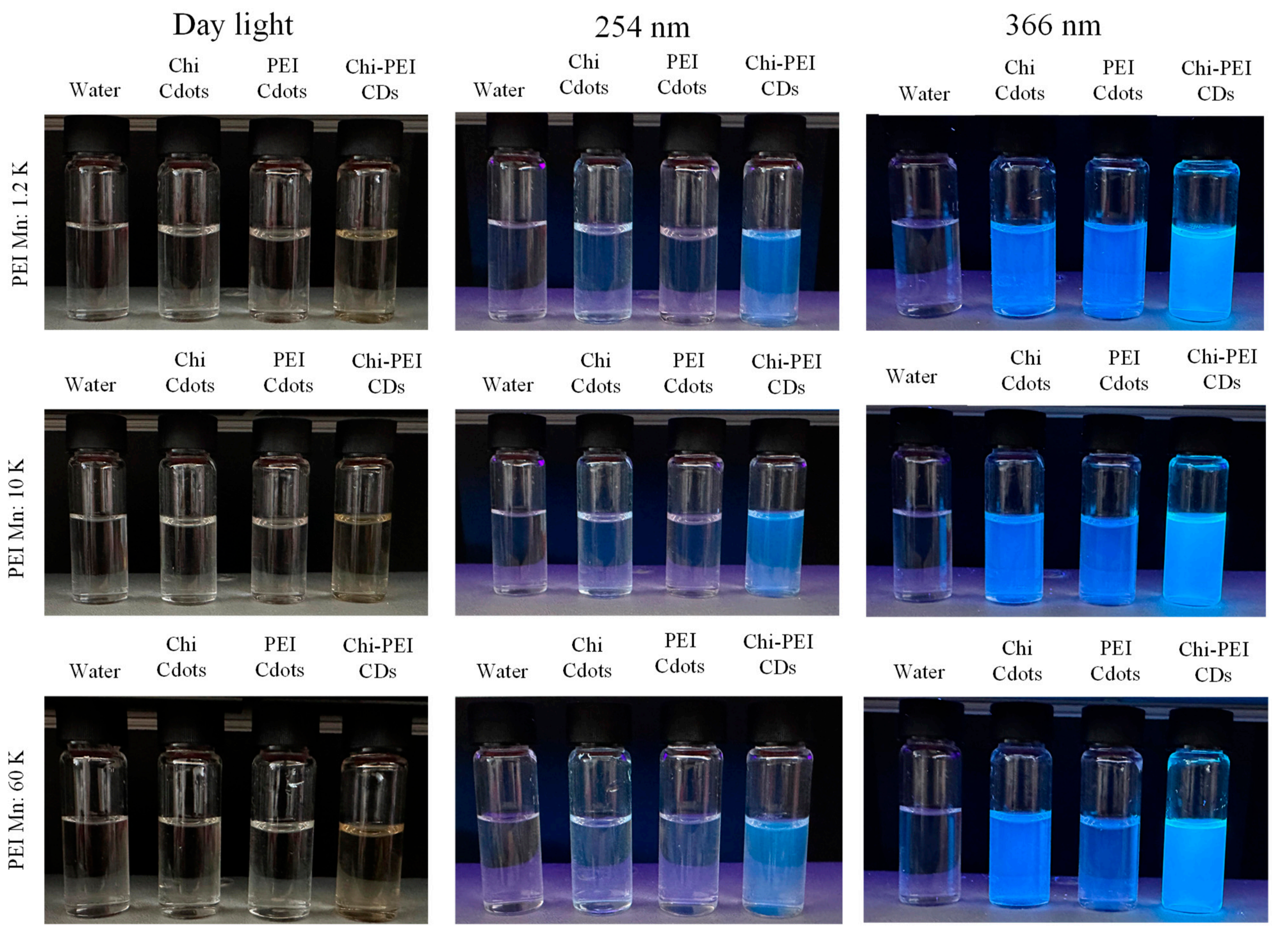

Figure S5. The digital camera images of prepared Cdots under day light, and 254 and 366 nm UV light.

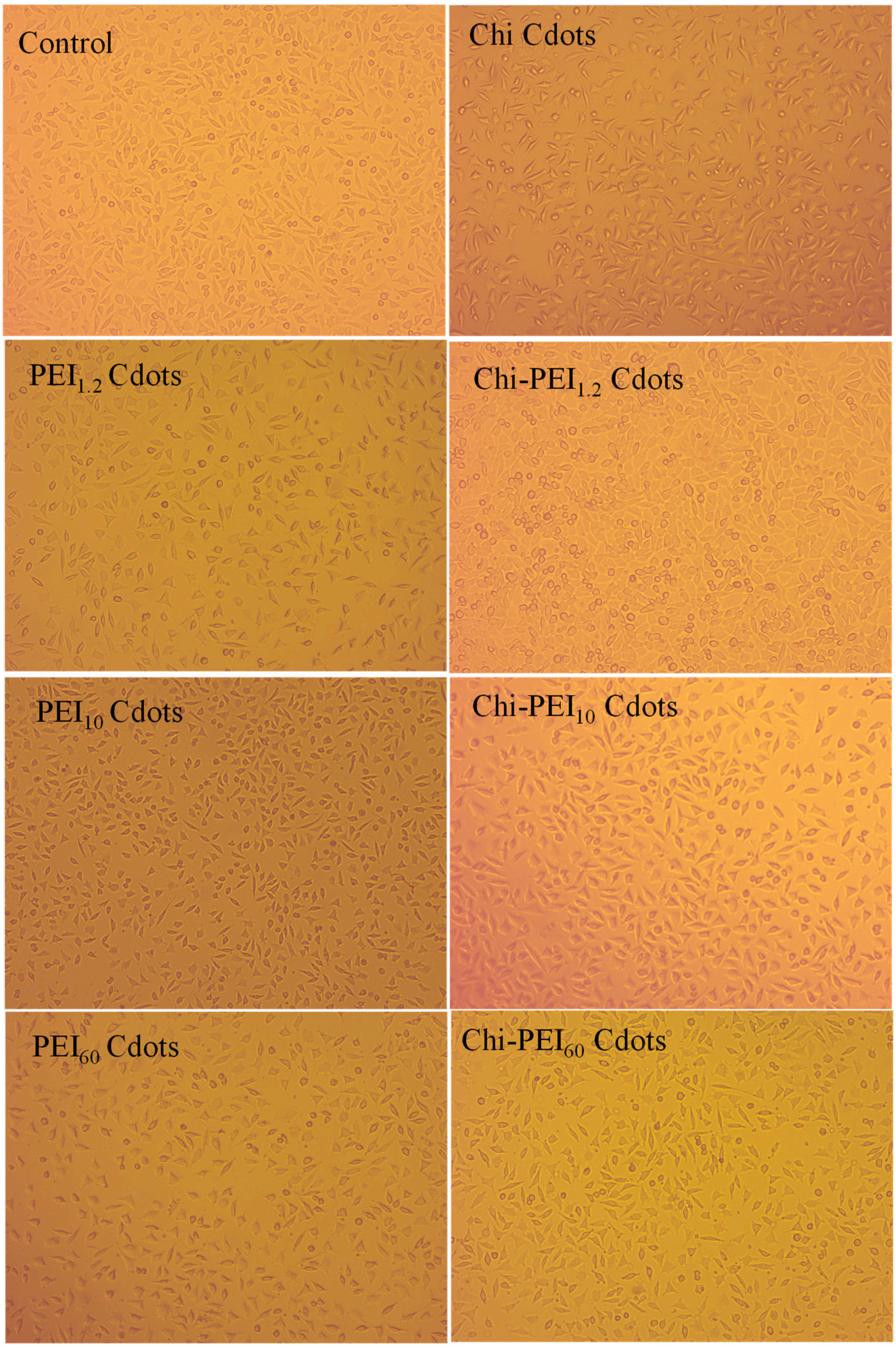

Figure S6. Optical microscope images of the L929 fibroblast cells used as the negative control group and in the presence of 1000 μg/mL concentrations of the Chi, PEI1.2, PEI10, PEI60, Chi-PEI1.2, Chi-PEI10, and Chi-PEI60 Cdots for a 24 h incubation time.
